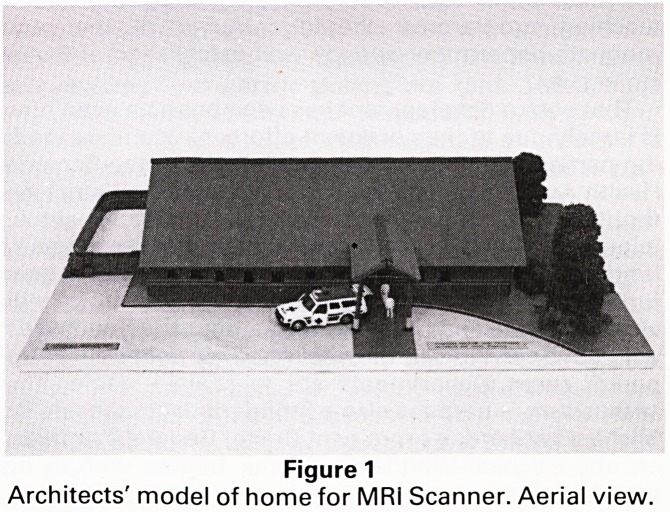# From Our Correspondents

**Published:** 1986-08

**Authors:** 


### From Our Correspondents

### Formation of New Department of General Practice in Bristol University

### 'Better Late than Never!'

So, at long last, Bristol University has created a full-time academic post in General Practice. Whilst the appointment of a Senior Lecturer in this field may not be recognised immediately by everyone as another giant leap for mankind,—coming as it does almost 30 years after the formation of the first of such departments in the United Kingdom—nonetheless, the elevation of local Principal, Dr. Michael Whitfield, to head the new department, marks a significant historical development in Bristol Medicine, which was becoming increasingly conspicuous and out-of-line in remaining virtually the last of our Medical schools without a single full-time position supporting the academic interests of the 1,700 G.Ps practising in the South West. The new department will be based initially at Canynge Hall, alongside the Department of Community Medicine, and its undergraduate teaching programme should complement the postgraduate department already well established at Exeter University.

That such a development has been possible even now, is largely due to the persistent efforts of countless working parties, together with the generosity of the Regional Health Authority, most of whose constituent District Authorities have agreed to prime the Trust fund set up jointly between these two Universities in order to secure funding over these next crucial few years. This has been further supported by numerous covenants from individual Doctors and practices throughout the South West who have recognised the vital need for continuing support if these Departments are to remain viable. This enthusiasm is perhaps also a fitting tribute to the late Dr. Michael Lennard, a prominent Bristol General Practitioner, and Michael Whitfield's former trainer, who in his time had done so much to promote this cause locally.

This is certainly not the place, nor indeed should there remain any further need to rehearse yet again the arguments supporting the rightful place of General Practice as a proper academic sub-discipline within Medicine, having its own particular body of knowledge, skills, research and literature. However, since our National Health Service presently relies on both Generalists and Specialists collaborating, each requires knowledge of the principles and experience of the practice of the others working methods. This should ideally be from an early stage in training, and the recently implemented revised Undergraduate Medical curriculum should encourage this. Moreover, in these times of virtually unprecedented financial stringency for the N.H.S, it not only makes sound educational sense for Generalists and Specialists to share their knowledge, experience and ideas, but also with the shifting emphasis of chronic care back into the Community—(what a deceptive phrase!)—we need to research well the cost-benefits of alternative methods of providing health care, other than our traditional institutionally-based ones. This will require a sound base for proper research to be conducted outside hospitals and institutions and the new Department of General Practice should form an ideal platform for this.

Unfortunately, however, even the Universities have been unable to remain immune to our financial malaise and ironically this new development comes just at the time when the recently published Mackenzie Report on General Practice in the United Kingdom Medical Schools, has highlighted the dire straits ('Money-for-Nothing?') in which most of these departments now find themselves.

However, since ours is the latest department to be established, we ought to be in a good position to learn from all the other mistakes, so that eventually we should have little excuse for not being at least among the best.

This will ultimately depend as much on Michael Whitfield's ability to harness sensitively the abundant enthusiasm, experience and skills of his local colleagues, as it will on his success in maintaining adequate funding for the new venture, which cannot and should not be expected to rely indefinately on G.P's continuing to dip into their own pockets in order to support and maintain their own Academic Presence—what other disciplines would expect to tolerate that? For, as Sir Raymond Hoffenberg said in commenting on the generally sad present state of affairs:—'These departments deserve better funding'.* It is up to us to see that they get it—at least it looks as if we should be in for some pretty exciting times ahead.

R. B. H. Maxwell

* Ref: B.M.J. 14th June 1986. pp 1545 and 1567.

### The Future of General Practice

When the Government publishes a Green Paper two and a half years later than promised and binds it in a blue cover you might be forgiven for thinking that there is confusion about; you may be right! Primary Health Care—an agenda for discussion finally saw the light of day in April 1986. It is designed to stimulate a review of the primary health services, the first time this has been done since the beginning of the National Health Service.

The primary health care services account for nearly a third of the total health expenditure and this has grown faster than the rest of the NHS. The expenditure covers the general practitioner services, dental and ophthalmic services and, most expensive of all, the pharmaceutical services. The Green Paper suggests ways of improving primary health care and some of these are very controversial.

The first of these proposes a Good Practice Allowance (GPA) and is designed to reward practices who practice high standard medicine. The main problem with this proposal is that this GPA is being equated with the consultants merit award scheme which has been previously rejected by general practitioners on the grounds that a secret assessment system is patently unfair and would be very difficult to administer. Most practitioners though, would approve an open system that rewards specific tasks that are recognised by the profession as being 'extra' to the normal job of a general practitioner. These include personal night cover, obstetric care, well-baby clinics and organised preventive care. Pricing such an award would be very difficult and would probably need to be introduced gradually.

The second main proposal relates to publicising the services that individual practices provide. Doctors have traditionally been hesitant to publicise their services but the government feels that patients need to know what there is on offer. There seems to be no good reason why the Family Practitioner Committee should not seek out information about the provision of special services within practices and publicise these in libraries and post offices in an enlarged Medical List. Whether it is appropriate to know the ages of the doctors or the fact that one offers acupuncture and another hypnosis remains to be seen.

One of the other controversial suggestions was that the capitation fee should assume a greater proportion of the doctors' income than the 40% at present. Most general practitioners are looking for smaller lists of patients and the idea put forward by the government of encouraging larger list sizes seems strange when the aim of the paper is to improve the service to patients. There are better ways to achieve good practice than simply rewarding doctors with large list sizes.

These and many other proposals on such areas as the wider role of the pharmacist, neighbourhood nursing schemes, information on referral rates, improving post-graduate educational uptake, the use of computers and so on will be debated in Local Medicine Committees throughout the country during this year. Hopefully the profession's response will enable the primary care services to advance but they will only do this if the government's response is seen as positive and not simply a further cost cutting exercise.

M. J. Whitfield

**Progress with the MRI Scanner**

On July 28th the building for the Bristol M.R.I. Scanner (Fig 1) is due to commence, on a site at Frenchay Hospital between the swimming pool and the adjacent green fields. It should be finished, and the Centre ready for its first patients by March 1987. By most standards this represents very satisfactory progress, and is due almost entirely to the further munificent generosity of the John James and Dawn James Trusts. Not only was the machine purchased (one million pounds) and donated to the Bristol Hospitals, but when delays seemed inevitable, they stepped in with a further outstanding loan to allow the building to start, and a further guarantee that will cover the first year's running costs. The total loan thus stands at over £600,000, and already in just over six months, £170,000 of this amount has been raised, but clearly much more needs to be done to achieve the target.

For the record, it should be recalled that the N.H.S. has been unable to help financially, although direct approaches were made both at Regional and D.H.A. levels. Their funds, quite understandably, were already committed, but their generosity in allowing the use of a piece of land at Frenchay Hospital is fully and gratefully acknowledged. In the official 'forward look' booklet issued from the S.W. Regional Health Authority, the installation of an M.R.I. Scanner in the south-west was anticipated in five years' time, so we have much to be thankful for in that this donation pre-empts official policy by quite a considerable span of time. Nevertheless the N.H.S. has indicated that by gradual involvement in the revenue consequences of the scanner, they will be fully responsible for the running and maintenance costs of the machine in five years' time. In the meantime this responsibility will devolve elsewhere.

The challenge was made, at the time of the original donation, to the citizens of Bristol, to raise the necessary money to finance the building and the initial revenue to run the machine. To this end a committee of Trustees was formed, with Dr. Terry Beddoe as chairman, Dr. Paul Goddard as the representative of the Bristol and Weston Authority, Dr. Clive Johnson of Southmead, and myself of Frenchay Health Authority. Considerable time and energy has been devoted to the cause and people have responded magnificently to the Appeal since it was announced. Money has come from almost every conceivable source, from individual donations to group activities; sponsored swims, walks or rides, horse jumping, boxing, parachuting, coffee mornings, bridge parties, jumble sales, raffles, snowmen, dances, skittle leagues, schools, Leagues of Friends, pubs, clubs and so on. The letters accompanying donations are amusing sometimes, heartwarming at others, but all times sincere, generous and encouraging, and ages have ranged from eight year olds to a ninety-two year old grateful patient. An article needs to be written on this subject alone, as it would reveal to the reader the best side of human nature. The man in the street can certainly rise to such a challenge! Through it all sounds so often, the racy tune of the Magnetic Appeal song composed by Paul Goddard, played and sung by our intrepid and magnificent Dr. Jazz Quartet. One felt from the start that we could not fail with such talent!

So now the scene is set. The machine is already under construction at the Picker International factory at Wembley. The building to house it is due to start (see photos of model). The three D.H.A.s are in agreement. The solicitors have signed. The Trustees are very grateful for all the support and encouragement proffered from so many people and sources. We look forward to the Spring.

J. L. G. Thompson

**Figure 1**
Architects' model of home for MRI Scanner. Aerial view.

**Random Reports from the Senior Scene**

Sadly, they didn't get a bite. Despite the high standard of the casting, and the swish of the fly line as it sailed through the air and uncurled in textbook fashion, the two intrepid anglers failed to reward the spectators with even the suggestion of a nibble. Nevertheless, this was of little surprise to the onlookers, as it is difficult to catch trout without a fly on the end of the line, no matter how beautifully it is dropped onto the green sward outside the Day Hospital. No, this was not a new form of physiotherapy, but a rather lighthearted interlude one lunch hour during the clinical examination that we hosted for the Diploma in Geriatric Medicine of the Royal College of Physicians! I didn't dare ask the candidates what they thought!

I recently had the privilege of examining in medical Finals in Belfast. This proved to be a most enjoyable occasion, and unlike our own Finals here in Bristol, one is paired, if one is a physician, with a surgeon as co-examiner. There was no shortage of patients in the surgical wards, or on their waiting lists, with conditions suitable for short cases. By the time the exam was over I had learned, or possibly re-learned, much of a surgical nature, and am now an expert on lumps in the scrotum. There do seem to be an awful lot of these in Northern Ireland! In all seriousness, however, it was a mutually edifying experience for surgeon and physician alike, and one which we all enjoyed.

While in Birmingham, both attending and participating in Professor Isaacs course on the Psychiatry of Old Age, the medal for the best presentation should undoubtedly have been given to Bernard himself. Although I can't quite remember how he related this story to the topic of the course, I feel it is worth retelling:

'An Englishman lost in the Irish countryside came upon a man cutting peat out of a bog, and asked him how long it would take to walk to the nearest town. The Irishman failed to reply. Thinking that he was probably deaf, the Englishman repeated his question in rather louder voice, only to meet with the same response. After a few minutes more, allowing what he considered to be long enough for even the most recalcitrant of neurones to function, our intrepid traveller repeated his question at full volume. Apart from the singing of the birds and the chortling of the crickets, silence continued to reign. With a shrug of his shoulders and an expletive or two, the enquirer moved off, but after he had travelled a few yards a thick Irish brogue shouted after him "twenty minutes". "What did you say?" yelled back the Englishman. "Twenty minutes". When asked by the frustrated and by now furious traveller as to why he hadn't volunteered that information a few minutes earlier, the reply came back "Well now, begorrah, praise the almighty, and surely to goodness, didn't I need to know how fast you was going to be walking?".'

This course is extremely worthwhile and good value to senior registrars or newly appointed consultants who feel they need to brush up on their knowledge in this field. And for anybody who needs to renew their stock of Irish jokes, it is clearly a must!

G. K. Wilcock

**Domestic differences: a pastoral solution**

There are times when it is not easy to communicate with one's dearly beloved offspring, or even with your wife. Such moments of domestic sadness and constraint often occur with surprising swiftness during the course of otherwise successful and vivacious conversation. These potentially dangerous family conversations are especially likely to occur when topics like teenage telephone bills, an unchanging menu of baked beans and their culinary merits, and the household heating (or its lack, or cost) come under general scrutiny by the family. A particularly dangerous place for such domestic talk to take place would seem to be in the kitchen.

For myself, it still seems amazing that there are so many different sides to these questions. All of them have a certain well-worn patina, due no doubt to long usage. It is not as if they were surfacing in the kitchen for the first time. Few in the family, with the exception of the turtoise, have not had their say many times before. The turtoise (see *Bristol Med.-Chir. Journal* 1984, **99**, 66) has proved singularly disappointing. It has not spoken about anything since first appearing in these columns.

However, here we are attempting to look at these matters in a rational way. In a modern family consensus rules, OK? The spark points seem to centre on certain limited subjects. These concern themselves with various people's abilities, or deficiences, in the fields of (1) fire lighting, (2) cooking, (3) domestic expenses, and (4) tidiness. Most people reading this journal, and still living with their families will appreciate the dangers of the topics.

So how to solve these domestic problems? The solution is quite straightforward. It ought, I suppose, to be pointed out that our family is not exactly entirely unanimous about the matter. However, I feel that other, less biased people reading this column might benefit from a solution.

Everything is really quite simple. It is clearly our own fault if we end up with problems which are so easily soluble. There seems little point in getting heated about inessentials. After all, gypsies have always known best. A horse and a wooden home on wheels are the obvious answers. I can't understand why the rest of the family doesn't want to COME AND LIVE IN A CARAVAN. It's generally cheaper, needs less heating and meals are not so formal. Also there is less need to put on appearances and there is not the necessity for immaculate tidiness. So there we are. No doubt, more domestic argument around the corner.

But maybe in the country the food will be smokier than the inside of the caravan itself, and anyway I expect there are rates to pay if you are not careful to move on from time to time. Who knows what the effect might be on your poor horse of a diet of baked beans, worries about the bills, muddle and no central heating. Thank goodness there are veterinary practitioners around to help with the horse's problems. Can anyone help us with our own?

J. D. Davies

## Figures and Tables

**Figure 1 f1:**